# The role of autophagy in cadmium-induced acute toxicity in glomerular mesangial cells and tracking polyubiquitination of cytoplasmic p53 as a biomarker

**DOI:** 10.1038/s12276-022-00782-4

**Published:** 2022-05-27

**Authors:** Ki-Tae Jung, Seon-Hee Oh

**Affiliations:** 1 Department of Anesthesiology and Pain Medicine, School of Medicine, Chosun University, 309 Pilmundaero, Dong-gu, Gwangju, 501-759 Korea; 2 School of Medicine, Chosun University, 309 Pilmundaero, Dong-gu, Gwangju, 501-759 Korea

**Keywords:** Macroautophagy, Diagnostic markers

## Abstract

Cadmium (Cd) is a highly toxic environmental pollutant that can severely damage the kidneys. Here, we show that Cd-induced apoptosis is promoted by the cytoplasmic polyubiquitination of p53 (polyUb-p53), which is regulated by the polyubiquitination of SQSTM1/p62 (polyUb-p62) and autophagy in mouse kidney mesangial cells (MES13E cells). p53 was detected in monomeric and different high-molecular-weight (HMW) forms after Cd exposure. Monomeric p53 levels decreased in a concentration- and time-dependent manner. HMW-p53 transiently accumulated in the cytoplasm independent of proteasome inhibition. The expression patterns of p53 were similar to those of p62 upon Cd exposure, and the interactions between polyUb-p53 and polyUb-p62 were observed using immunoprecipitation. P62 knockdown reduced polyUb-p53 and upregulated nuclear monomeric p53, whereas p53 knockdown reduced polyUb-p62. Autophagy inhibition induced by *ATG5* knockdown reduced Cd-induced polyUb-p62 and polyUb-p53 but upregulated the levels of nuclear p53. Pharmacological inhibition of autophagy by bafilomycin A1 increased polyUb-p62 and polyUb-p53 in the cytoplasm, indicating that p53 protein levels and subcellular localization were regulated by polyUb-p62 and autophagy. Immunoprecipitation and immunofluorescence revealed an interaction between p53 and LC3B, indicating that p53 was taken up by autophagosomes. Cd-resistant RMES13E cells and kidney tissues from mice continuously injected with Cd had reduced polyUb-p53, polyUb-p62, and autophagy levels. Similar results were observed in renal cell carcinoma cell lines. These results indicate that cytoplasmic polyUb-p53 is a potential biomarker for Cd-induced acute toxicity in mesangial cells. In addition, upregulation of nuclear p53 may protect cells against Cd cytotoxicity, but abnormal p53 accumulation may contribute to tumor development.

## Toxicology: A cellular signature of cadmium poisoning

The cellular localization and chemical modification of a protein that acts as a critical safeguard against cellular damage may directly contribute to the toxic effects of cadmium. P53 is an essential tumor suppressor that is also involved in numerous other important biological functions. Ki-Tae Jung and Seon-Hee Oh of Chosun University, Gwangju, South Korea have now demonstrated that this protein also undergoes rapid changes in response to the environmental pollutant cadmium. P53 normally manages gene expression in the nucleus, but the authors found that it is rapidly shuttled to the cytoplasm and subjected to extensive chemical modification in cadmium-treated cultured kidney cells. This relocation appears to contribute directly to subsequent cell death, and the authors suggest that this P53 response could be an important biomarker for diagnosing human cadmium exposure.

## Graphical abstract

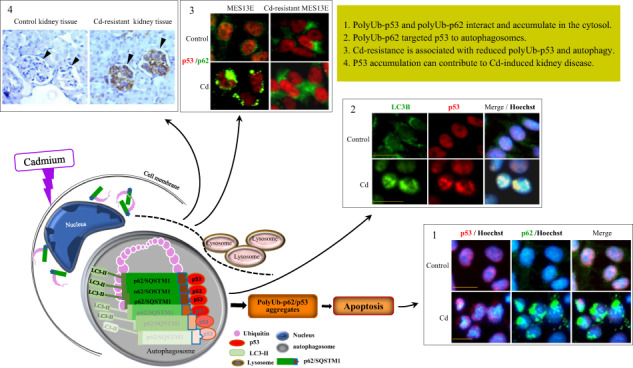

## Introduction

Cadmium (Cd) is a global environmental pollutant that can cause various pathophysiological conditions in humans depending on the duration and level of exposure. In addition to being acutely toxic to the liver and lungs, Cd can accumulate in the human body during chronic exposure, which can cause age-related diseases, such as renal dysfunction^1^. After absorption, Cd binds to metallothionein (Cd-MT) in the liver and is transported to the kidneys, where it is filtered through the glomeruli and reabsorbed by proximal tubular cells, after which it can accumulate in the kidneys. Therefore, most studies on the toxic effects of Cd in the kidneys have focused on proximal tubular cells. Kidney damage is caused by tubular and glomerular dysfunction^2^; thus, Cd-induced glomerular toxicity should be clarified to better understand Cd-induced kidney damage.

The tumor suppressor p53 functions as a transcription factor that regulates numerous cellular processes, including apoptosis. p53 plays an important role in determining cell survival or cell death through DNA repair or apoptosis, depending on the extent of DNA damage^3^. Thus, p53 protein levels are critical factors for cellular function. Normally, p53 protein is maintained at a very low level. However, under stress, p53 protein levels are increased and stabilized via various posttranslational modifications, including methylation, acetylation, phosphorylation, and sumoylation^4^. In contrast, p53 protein levels are negatively regulated by the ubiquitin–proteasome pathway, which is modulated by various E3 ligases, including mouse double minute 2 homolog (MDM2)^5^. The involvement of p53 in Cd-induced toxicity has been reported in human (HK-2) and rat (NRK52E) proximal tubular epithelial cells and mouse kidneys. Cd increases p53 levels by repressing the transcription of ubiquitin-conjugating enzyme 2D (UBE2D) without changing p53 gene expression or proteasome activity^6,7^. p53 accumulates in Cd-exposed HK-2 cells by cooperating with the Notch1 and phosphatidylinositol-3-kinase (PI3K)/Akt signaling pathways^8^. Cd-induced increases in p53 contribute to apoptosis and cell cycle arrest via activation of the downstream targets p21 and p27^9^. In addition to transcriptional regulation, p53 induces apoptosis by directly targeting the mitochondria^10^, suggesting that the subcellular localization of p53 can play an important role in p53-mediated apoptosis.

Autophagy is a lysosomal degradation pathway that is essential for cellular function and survival because it maintains the quality control of proteins and organelles^11^. Autophagy is implicated in kidney damage, but its role is still under debate. The protective role of autophagy against Cd-induced apoptosis has been reported in rat kidney tubular cells and rat renal mesangial cells^12–14^. In contrast, Cd-induced autophagy induced by glycogen synthase kinase 3β (GSK3β) and calcium-mediated extracellular signal-regulated kinase (ERK) activation has been associated with toxic effects in mouse glomerular mesangial cells^15,16^. This discrepancy may be explained by the differences in species. Therefore, more detailed studies are required to determine the effect of Cd-induced autophagy on kidney damage.

Previous studies have shown that p53 downregulation by autophagy promotes tumor survival^17–19^. p53 regulates autophagy differently depending on its intracellular localization. Nuclear p53 can induce autophagy by upregulating DRAM (damage-regulated autophagy modulator) and sestrin-1/sestrin-2 transcription^20,21^, and cytoplasmic p53 can repress autophagy by activating mammalian target of rapamycin (mTOR)^22,23^. p53 is degraded via transglutaminase 2 (TGase 2)-mediated autophagy in renal cell carcinoma (RCC) cells, promoting tumor formation^18^. Considering the established role of p53 in Cd-induced kidney damage, autophagy may be associated with p53-mediated apoptosis. However, the relationship between autophagy and p53 in Cd-induced kidney toxicity in normal cells remains elusive.

This study aimed to explore the regulatory mechanisms of p53 associated with autophagy and the role of p53 in renal toxicity induced by Cd. We used mouse glomerular mesangial cells, which support the structure of glomerular capillaries and regulate glomerular filtration. Mesangial cells are in direct contact with capillary endothelial cells and can exchange signals and substances directly with them^24^. Therefore, to better understand Cd-induced renal toxicity, the role of mesangial cells needs to be further investigated.

## Materials and methods

### Reagents and antibodies

Cadmium acetate (289159), 3-(4,5-dimethylthiazol-2-yl)-2,5-diphenyltetrazolium bromide (MTT) (M2128), MG132 (M8699), Hoechst 33342 (B2261), bafilomycin A1 (B1793), and an anti-β-actin antibody (ab-8226) were obtained from Sigma-Aldrich. SQSTM1/p62 (sc-25575), p21 (sc-6246), ubiquitin (sc-8017), rhodamine-conjugated goat anti-rabbit (sc-2091), and FITC-conjugated goat anti-mouse (sc-2010) antibodies were purchased from Santa Cruz. PARP-1 (9532), procaspase-3 (9662), cleaved caspase-3 (9661), p53 (2524), phospho-p53 (S15, 9284), acetyl-p53 (Lys379, 2570), LC3B (2775, 3868), p27 (2552), and phospho-γ‐H2AX (S139, 2577) antibodies were purchased from Cell Signaling. APG3L (AP1807a) and QSTM1/p62 (H00008878-M01) antibodies were purchased from Abgent and Abnova, respectively. An MDM2 antibody was purchased from GenTex (100653).

### Cell culture

MES13E mouse kidney mesangial cells (CRL-1927™) were obtained from the American Type Culture Collection (Rockville, MD, USA, ATCC). The human RCC cell lines Caki-1 and ACHN were obtained from Dr. Soo-Youl Kim, Division of Cancer Biology, National Cancer Center, Republic of Korea. The cell lines were maintained in Dulbecco’s modified Eagle’s medium (WelGene, LM-001-05) supplemented with 10% fetal bovine serum (WelGene, S001) and penicillin–streptomycin (WelGene, LS203). The cells were cultured at 37 °C in a humidified incubator with 5% CO_2_. All cell lines were examined and determined to be mycoplasma-free and were used between passages 3 and 12.

### Cytotoxicity assays

Cell viability was analyzed using an MTT assay. Cell suspensions (180 μL, 1 × 10^5^ cells/mL) were seeded in a 48-well plate and cultured for 2 days. After chemical treatment with MTT (0.5 mg/mL), the plate was incubated for 2 h at 37 °C. The absorbance of formazan crystals dissolved in dimethyl sulfoxide was measured using a microplate reader (PerkinElmer) at 540 nm. Each experiment was repeated at least three times. The values are presented as the mean ± standard deviation (SD) of the fold increase compared to the control.

### Transfection

Cells were seeded into 6-well plates (2.5 × 10^5^ cells) and transfected with 20 pmol of siRNA using Lipofectamine^TM^ RNAiMAX reagent (Invitrogen, 56531) according to the manufacturer’s instructions. The medium was replaced with fresh complete medium after 6 h, and cells were continuously cultured for an additional day before treatment. The target sequences of the siRNA oligos were as follows: 5′-GAGUCAGCUAUUUGACGUU-3′ for Atg5, 5′-CUUGUAGUUGCAUCACGUA-3′ for p62, and 5′-GUCUGUUAUGUGCACGUAC-3′ for p53 (synthesized by Bioneer, Daejeon, South Korea). Nonspecific siRNA was purchased from Sigma-Aldrich (St. Louis, MO, SIC001).

### Immunoblotting and immunoprecipitation (IP)

Cells were lysed in lysis buffer (Cell Signaling, 9803) supplemented with a protease cocktail (Roche, 04693132001). Proteins (15–35 μg) were resolved using 10–12% SDS–PAGE and transferred to polyvinylidene difluoride (PVDF) membranes (Millipore, IPVH00010). The membranes were blocked with 5% skim milk (Bioshop, SK1400) and then probed with primary and secondary antibodies. The proteins were visualized using a chemiluminescent substrate (Millipore, WBKLS0100). For IP, cells were lysed in lysis buffer (0.05 M Tris-HCl [pH 7.4], 250 mM NaCl, 0.25% Triton X-100, 10% glycerol) with protease cocktail. Total proteins (600 μg) were precleared using 50% protein G Plus-agarose beads (Santa Cruz, sc-2002) and centrifuged at 12,000 × *g* for 10 min at 4 °C. The supernatants were incubated with the indicated primary antibody, rabbit IgG (Sigma-Aldrich, 12-370), or mouse IgG (Sigma-Aldrich, 12-371) overnight at 4 °C. The immunocomplexes were captured using protein G Plus-agarose beads and washed with ice-cold PBS several times. The washed beads were resuspended in 2X Laemmli loading buffer and boiled, and then the proteins were eluted and processed for immunoblotting. The band intensities were quantified using ImageJ software.

### Immunofluorescence (IF)

After culturing cells on a cover slip (Marienfeld, D111580), the cells were fixed in neutral-buffered formalin (NBF, Sigma-Aldrich, HT501128) for 10 min on ice. The cells were then washed with PBS and treated with 0.05% Triton X-100 (Sigma, T8787) for 20 min. After washing with PBS, the cells were blocked with 2% bovine serum albumin (Bioshop, ALB001). The cells were then incubated with primary antibodies and fluorescently conjugated secondary antibodies. The nuclei were counterstained with Hoechst 33342 (1 μg/mL), and images were captured using a Nikon Eclipse TE300 fluorescence microscope.

### Immunohistochemistry (IHC)

The tissues fixed in NBF were embedded in paraffin. Sections (4-μm thick) were subjected to antigen retrieval in 0.01 M sodium citrate buffer (pH 6). After removing the endogenous peroxidase activity using 0.3% H_2_O_2_, the sections were incubated with a p53 antibody (Santa Cruz, 1:50) overnight at 4 °C. Negative controls were performed for each specimen without the primary antibody. A Polink-2 AP broad detection kit (GBI Labs, D68-18) was used according to the manufacturer’s protocol. After counterstaining with hematoxylin, the sections were mounted using Simpo-mount (GBI Labs, E01-18).

### Animal experiments and Cd injection

The animal care and Cd injection procedures were performed as previously described^25^. Briefly, 6-week-old male C57BL/6 (C57) mice (Orientbio, Seongnam, South Korea) were kept under regular conditions with 12 h light-dark cycles at 50–60% humidity. The animals were randomly divided into two groups of eight mice each. The mice were treated with cadmium acetate (1 mg/kg body weight) or an equivalent volume of saline via intraperitoneal (i.p.) injections for 20 weeks. The animals were then anesthetized with 5% isoflurane in oxygen. The animal experiments were conducted with the permission of the Chosun University Animal Protection and Utilization Committee (IACUC) (No. CIACUC2019-A0045).

### Statistical analysis

All experiments were performed independently at least three times. The data are presented as the mean ± standard deviation (SD). The statistical significance of differences between experimental groups was determined using one-way ANOVA. *P* values <0.05 were considered to indicate statistical significance.

## Results

### Expression of p53 in response to Cd in MES13 cells

**Fig. 1:**
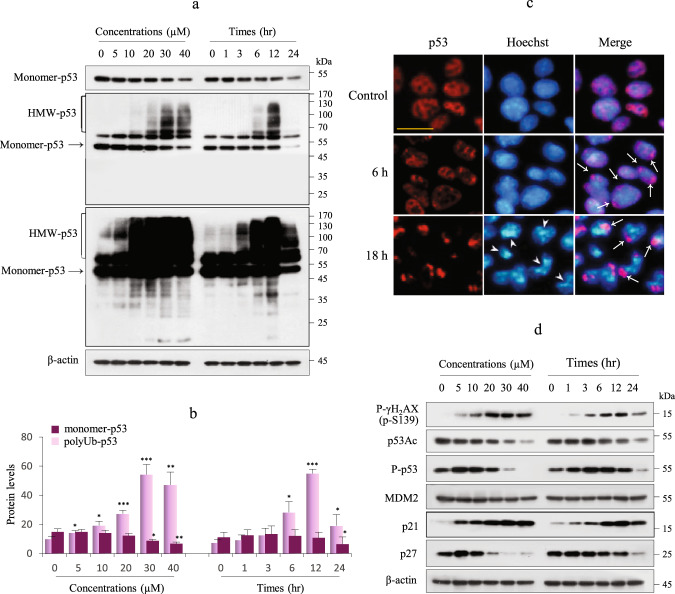
Expression of p53 and its downstream targets in Cd-exposed MES13E cells. **a**, **b** Cells were treated with increasing Cd concentrations for 18 h or with 23 µM Cd for up to 24 h. The p53 protein was analyzed by immunoblotting. The levels of HMW-p53 and monomer-p53 were quantified by densitometry and normalized to those of β-actin. The data are presented as the mean ± standard error of the mean (SEM) (*n* = 3). *P < 0.05; **P < 0.005; ***P < 0.0005. **c** IF staining for p53 (red) was performed in cells treated with Cd for 6 h and 18 h as described in the “Materials and methods” section. The nuclei were counterstained with Hoechst 33342 (blue), and images were acquired with a fluorescence microscope. Scale bar = 25 µm. **d** Cells were treated as described in (**a**). Immunoblotting was performed for the indicated proteins. β-Actin was used as the loading control, *n* = 3.

Previously, we found that Cd exposure of MES13E cells induced apoptosis via caspase-dependent poly-(ADP ribose) polymerase 1 (PARP-1) cleavage^26^. To determine the response of the p53 tumor suppressor protein during Cd-induced apoptosis, MES13E cells were exposed to gradually increasing concentrations of Cd for 18 h or exposed to the half-maximal inhibitory concentration (IC_50_) of Cd, ~23 μM, for varying durations. To determine whether the sensitivity to Cd was associated with p53, we first investigated p53 protein levels using immunoblotting. MES13E cells had a high basal level of p53, which was expressed in both monomeric and multiple high-molecular-weight (HMW) forms, in response to Cd. The levels of monomeric p53 protein (monomer-p53) decreased in a concentration- and exposure time-dependent manner. In contrast, HMW-p53 levels continuously increased as the Cd concentration increased up to 30 µM Cd but slightly decreased with the 40 µM Cd treatment. The number of bands below monomer-p53 was low compared to the number of HMW-p53 bands above 55 kDa, indicating that p53 degradation was low. In the time course of the experiments, HMW-p53 levels peaked at 12 h of Cd treatment and then decreased afterward (Fig. 1a, b). The subcellular localization of p53 was examined using IF. In unstimulated control cells, p53 had a predominantly nuclear distribution. However, Cd exposure resulted in changes in the p53 subcellular distribution. At 6 h of Cd exposure, p53 aggregated in the perinuclear area (arrows), and it was heavily aggregated in the cytoplasm at 18 h; this aggregation was accompanied by nuclear shape changes and chromatin condensation (arrowheads) (Fig. 1c). Changes in the subcellular localization of p53 after Cd exposure were further confirmed by fractionation studies. After 6 h of Cd exposure, p53 and polyUb-p53 levels were significantly increased in the cytoplasmic fraction (Supplementary Fig. 1).

p53 is activated in response to DNA damage and leads to cell cycle arrest and apoptotic cell death. Phosphorylation of gamma-H2A family member X (p-γH2AX), a marker of double-stranded DNA breakage, was induced at 1 h after Cd exposure at all the Cd doses used. p53 function is associated with the levels of the p53 protein, which is regulated by various posttranscriptional modifications. Thus, we evaluated the levels of acetylated p53 (p53Ac, Lys379) and phosphorylated p53 (p-p53, Ser15) using immunoblotting with specific antibodies in Cd-exposed MES13E cells. The changes in p53Ac were consistent with the changes in monomer-p53. P-p53 levels increased with low Cd concentrations and short incubation times and decreased in parallel with HMW-p53 levels, indicating that p53 activation may be inhibited by HMW-p53. Next, we examined the expression of two well-known p53 target genes, *MDM2* and the *cyclin-dependent kinase inhibitor p21* (*p21*^*Waf1*^). MDM2 levels did not change following Cd exposure. p21 levels increased in a dose- and exposure time-dependent manner, indicating that the Cd-induced upregulation of p21 is independent of p53 transcriptional activity. In contrast, p27 (p27^Kip1^) levels were positively correlated with p53 levels (Fig. 1d, Supplementary Fig. 2). In addition, Cd sensitivity was related to apoptosis induced by caspase-8, caspase-3, and poly-ADP ribose polymerase 1 (PARP-1) cleavage (Supplementary Fig. 3a, b). Collectively, these findings indicate that Cd-mediated HMW-p53 induction results in cytoplasmic aggregation, which may be associated with unknown functions of p53. In addition, Cd exposure may result in cell cycle arrest through p53-independent induction of p21 in response to DNA damage and apoptosis.

### p53 interacts with ubiquitin, and its degradation is independent of the proteasome

**Fig. 2:**
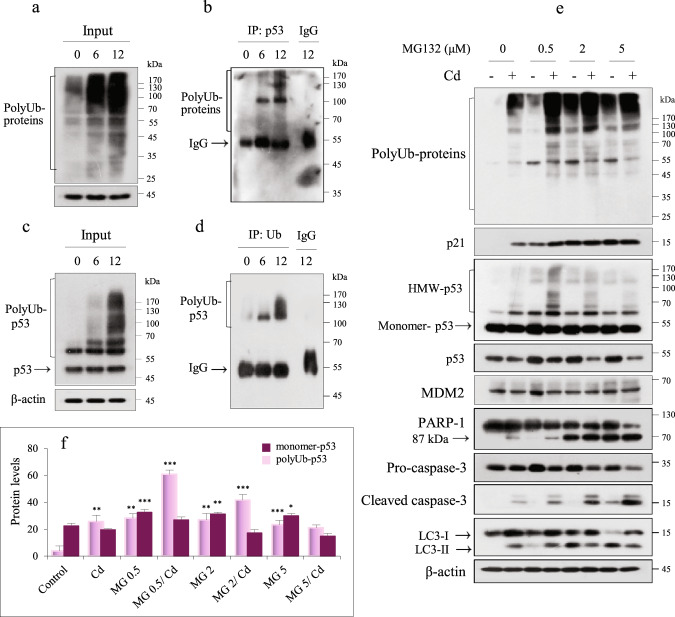
Accumulation of polyUb-p53 during Cd exposure is independent of proteasomes. **a**–**d** Cells were treated with 23 µM Cd for 6 h and 12 h. Immunoblotting was performed on lysates for Ub, and 600 µg of remaining protein was used for IP analysis with a p53 antibody and mouse IgG and then immunoblotted for Ub (**a**, **b**), and vice versa (**c**, **d**). **e**, **f** MES13 cells were pretreated with increasing concentrations of MG132 (0.5–5 µM) for 2 h and then treated with Cd (23 µM) for 12 h, harvested, lysed, and immunoblotted for the indicated proteins. β-Actin was used as the loading control. The levels of HMW-p53 and monomer-p53 were quantified by densitometry and normalized to those of β-actin. The data are presented as the mean ± SD (*n* = 3). *P < 0.05; **P < 0.005; ***P < 0.0005.

p53 expression is tightly controlled via the ubiquitin (Ub)-proteasome degradation system. To examine whether HMW-p53 was associated with polyubiquitination (polyUb), IP was performed to detect p53 in cell lysates treated with Cd for 6 or 12 h, after which immunoblotting was performed with an anti-Ub antibody (Fig. 2a, b). IP analysis was also performed by detecting Ub in the same protein lysates and was followed by immunoblotting with an anti-p53 antibody (Fig. 2c, d). These results confirmed an interaction between p53 and Ub. Furthermore, these results suggested that the p53 protein levels may be dependent on the Ub-proteasome degradation system. To confirm this, cells were exposed to different concentrations of MG132, a proteasome inhibitor. Cells treated with MG132 with or without Cd had increased p21 protein levels (Fig. 2e), indicating that MG132 effectively inhibited proteasomes. Treatment with MG132 alone resulted in polyUb protein accumulation compared to the situation in control cells. PolyUb protein levels were further increased in Cd-exposed cells, and this effect was exacerbated in cells treated with low concentrations of MG132. Treatment with MG132 alone increased monomer-p53 levels but did not increase HMW-p53 levels. In MG132- and Cd-treated cells, an increase in HMW-p53 was noticeable at low concentrations of MG132 (≤0.5 μM), but the level of HMW-p53 decreased at high concentrations of MG132 (Fig. 2e, f, Supplementary Fig. 4a), suggesting that the expression of polyUb-p53 in Cd-exposed MES13E cells is partially dependent on the proteasome. The expression pattern of HMW-p53 in MG132- and Cd-treated cells was similar to that of polyUb-p62 expression (Supplementary Fig. 4b). p53 stability may be controlled by MDM2, a p53-specific E3 ubiquitin ligase^27^. MG132 treatment with or without Cd did not affect the levels of MDM2. Inhibiting proteasomes induced apoptosis through caspase-3 and PARP-1 cleavage in a concentration-dependent manner, which was further enhanced in Cd-exposed cells. MG132 alone increased the levels of the microtubule-associated protein 1A/1B light chain 3 B (LC3-II), a component of autophagy, in the treated cells compared to the control cells. However, the exposure of MG132-pretreated cells to Cd did not increase the LC3-II level but rather decreased it in an MG132 concentration-dependent manner (Fig. 2e), indicating that autophagy did not play a compensatory role in proteasome blockade. To confirm this, we investigated the effect of MG132 on p53 in cells genetically deficient in the autophagy-related gene *ATG5*. Compared to *ATG5*-knockdown cells, ATG5-knockdown cells treated with MG132 (0.5 μM) also showed no significant changes in the levels of polyUb-p62, polyUb-p53, and polyUb-proteins in response to Cd (Supplementary Fig. 4c). These results indicated that the levels of monomer- and polyUb-p53 protein in Cd-exposed MES13E cells were dependent on polyUb-p62 and autophagy. However, we did not determine how proteasome blockade induced autophagy inhibition in Cd-exposed cells.

### PolyUb-p53 interacts with polyUb-p62 in Cd-exposed MES13E cells

**Fig. 3:**
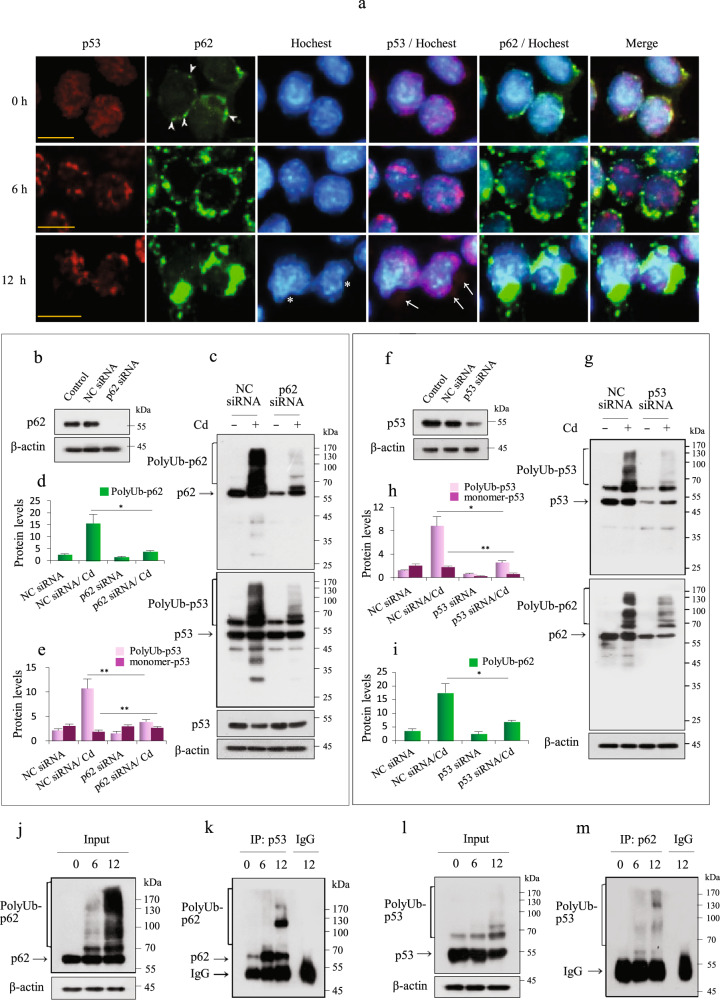
PolyUb-p53 interacts with polyUb-p62 in Cd-exposed MES13E cells. **a** Cells cultured on coverslips were treated with 23 µM Cd for 6 h and 12 h and fixed, and then IF was performed for both p53 (red) and p62 (green). Nuclei were counterstained with Hoechst 33342 (blue), and images were acquired with a fluorescence microscope. Scale bar = 25 µm. **b** The KD efficiency for *p62*-specific siRNA at 24 h of transfection was evaluated by immunoblotting for p62. NC = negative control. **c** Cells transfected with NC and *p62* siRNA were exposed to 23 µM Cd for 12 h, and the lysates were analyzed for the indicated proteins by immunoblotting. **d**, **e** The levels of polyUb-p62, polyUb-p53, and monomeric p53 were quantified by densitometry and normalized to those of β-actin. The data are presented as the mean ± SD (*n* = 3). **f** The KD efficiency of *p53*-specific siRNA at 24 h of transfection was evaluated by immunoblotting for p53. NC = negative control. **g** Cells transfected with NC and *p53* siRNA were exposed to 23 µM Cd for 12 h, and the lysates were analyzed for the indicated proteins by immunoblotting. **h**, **i** The levels of polyUb-p62, polyUb-p53, and monomer-p53 were quantified by densitometry and normalized to those of β-actin. The data are presented as the mean ± SD (*n* = 3). **j**–**m** Cells were treated with 23 µM Cd for 6 h and 12 h. Immunoblotting was performed for Ub, where IP was performed with 600 µg of the remaining protein with p53 antibody, followed by immunoblotting with p62 antibody (**j**, **k**) or vice versa (**l**, **m**). *P < 0.05; **P < 0.005.

We found that proteasome inhibition did not lead to an accumulation of p53 protein in Cd-exposed MES13E cells (Fig. 2e). Therefore, we examined the involvement of autophagy in regulating p53 multimerization, ubiquitination, and localization. We first examined the involvement of p62, an autophagic cargo adapter. Previously, we found that p62 was expressed in both monomeric and polyUb-conjugated forms in Cd-exposed MES13 cells^26^. The number of bands below 55 kDa, showing degraded ubiquitinated p62, was markedly lower than the number of bands of polyUb-p62 above 55 kDa, indicating that autophagic flux was limited or occurred slowly. Despite the increasing LC3-II levels, cytosolic LC3-I consistently increased with Cd treatment in a dose- and exposure time-dependent manner (Supplementary Fig. 3c). Immunoblotting for p53 showed a pattern similar to that for p62 (Fig. 1a, Supplementary Fig. 3c), as polyUb-p53 and polyUb-p62 transiently increased during Cd exposure, and the levels of both proteins began to decrease after 24 h of Cd exposure (Supplementary Fig. 3b). These results showed a possible interaction between p53 and p62 in Cd-exposed cells. To address this interaction, we first performed IF using anti-p53 and anti-p62 antibodies. In untreated control cells, p53 was prominently distributed in the nucleus. After 6 h of Cd exposure, p53 began to aggregate and was gradually redistributed to the perinuclear area. After 12 h, p53 aggregated in the periphery of the nucleus and in the cytoplasm (arrows). In untreated control cells, the p62 signal was low but widespread throughout the nucleus and was punctate in the perinuclear area (arrowheads). After 6 h of Cd exposure, the p62 signal increased, was distinctly localized in the perinuclear area and cytoplasm, and eventually aggregated only in the cytoplasm, merging with the cytoplasmic p53 signal. Chromatin became condensed, and the nuclei had a concave shape in Cd-exposed cells (Fig. 3a, asterisk). To further test p53 and p62 interactions, we knocked down *p62* using siRNA. p62 suppression markedly reduced the levels of monomer-p62 and polyUb-p62. p62 reduction also suppressed polyUb-p53 levels and recovered the monomer-p53 levels in response to Cd exposure (Fig. 3b–e). In turn, knockdown of p53 reduced the levels of monomer-p62 and polyUb-p62 (Fig. 3f–i). This relationship between p53 and p62 was confirmed using immunofluorescence. Genetic silencing of *p62* increased the nuclear p53 signal, and *p53* silencing reduced p62 aggregation in the cytoplasm (Supplementary Fig. 5). The interaction between both proteins was further confirmed using IP analysis. p53 and p62 immunocomplexes from lysates of Cd-exposed cells were subjected to immunoblotting using anti-p62 and anti-p53 antibodies, respectively. Both results showed an interaction between p53 and p62 (Fig. 3j–m). Therefore, these results indicate that polyUb-p53 interacts with polyUb-p62 in the cytoplasm.

### Subcellular localization of polyUb-p53 is regulated by autophagy

**Fig. 4:**
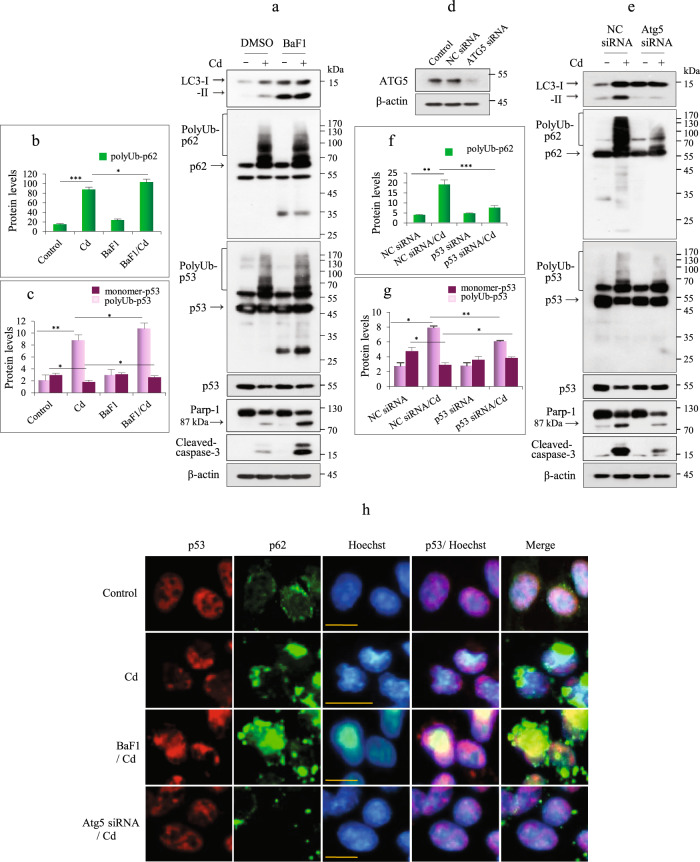
PolyUb-p53 levels were regulated by autophagy. **a** Cells were exposed to 23 µM Cd for 12 h with or without pretreatment with 20 nM BaF1 for 2 h. The lysates were subjected to immunoblotting for the indicated proteins. **b**, **c** The levels of polyUb-p62, polyUb-p53, and monomer-p53 were quantified by densitometry and normalized to those of β-actin. The data are presented as the mean ± SD (*n* = 3). **d** The KD efficiency for *ATG5*-specific siRNA was evaluated by immunoblotting for Atg5. NC = negative control. **e** Lysates were analyzed for the indicated proteins by immunoblotting. **f**, **g** The levels of polyUb-p62, polyUb-p53, and monomer-p53 were quantified by densitometry and normalized to those of β-actin. The data are presented as the mean ± SD (*n* = 3). **h** Cells cultured on coverslips were treated with 23 µM Cd and fixed, and then IF was performed for both p53 (red) and p62 (green). The nuclei were counterstained with Hoechst 33342 (blue). Scale bar = 25 µm. *P < 0.05; **P < 0.005; ***P < 0.0005.

Autophagosome contents are degraded by lysosomal enzymes. Since p62 is a cargo adapter, we tested whether p62 is involved in the autophagosomal uptake of p53. First, we inhibited autophagic flux using bafilomycin A1 (BaF1), an inhibitor of fusion between autophagosomes and lysosomes. BaF1 pretreatment before Cd exposure upregulated LC3-II, polyUb-p62, monomer-p62, polyUb-p53, and monomer-p53 levels compared to those in Cd-treated cells without BaF1 (Fig. 4a–c). Consistently, BaF1 treatment increased the p53 signal in the nucleus and cytoplasm and further increased the aggregation of p62 in the cytoplasm (Fig. 4h). In addition, BaF1 treatment enhanced Cd-induced caspase-3 and PARP-1 cleavage (Fig. 4a). These results were confirmed by silencing *autophagy-related gene 5* (*ATG5*). The efficiency of *ATG5* knockdown was evaluated by measuring the protein levels of ATG5 and LC3-II. Compared to Cd-exposed negative control (NC) siRNA-transfected cells, Cd-exposed cells transfected with *ATG5* siRNA exhibited reduced polyUb-p62 and polyUb-p53 levels, increased monomer-p53 levels, and reduced cleaved procaspase-3 and PARP-1 levels (Fig. 4d–g). In Cd-exposed *ATG5*-knockdown cells, p53 was primarily distributed in the nucleus, similar to the case in control cells. Consistent with the data from the immunoblotting experiments, p62 was suppressed by *ATG5* knockdown (Fig. 4h). These results suggest that the accumulation of cytoplasmic p53 may be associated with autophagy in Cd-exposed cells.

### PolyUb-p53 is upregulated in autophagosomes

**Fig. 5:**
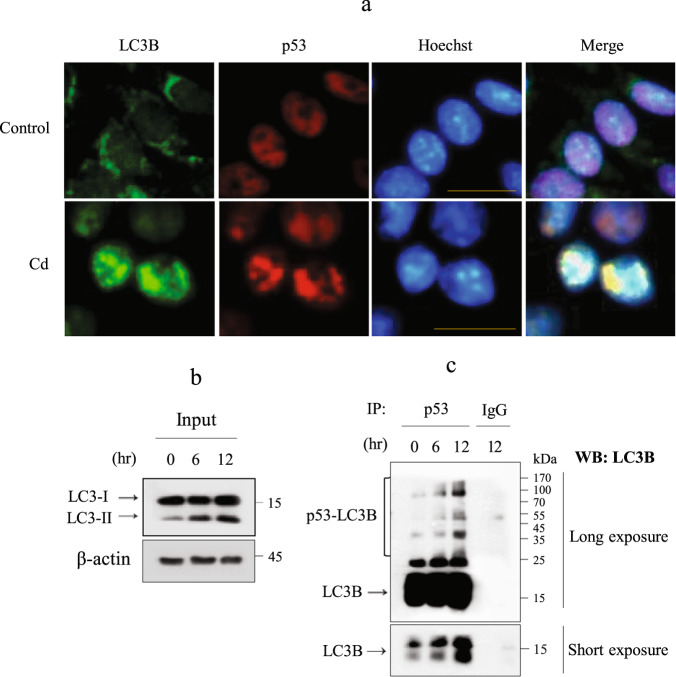
p53 colocalized with LC3B. **a** Cells cultured on coverslips were treated with 23 µM Cd for 12 h, fixed, and then subjected to IF for both p53 (red) and LC3B (green). Nuclei were counterstained with Hoechst 33342 (blue). Scale bar = 25 µm. **b**, **c** Cells were treated with 23 µM Cd for 6 h and 12 h. The lysates were immunoblotted for LC3B, and 600 µg of the remaining protein was analyzed by IP with a p53 antibody and mouse IgG. Then, immunoblotting was performed with a LC3B antibody.

The above results revealed that the levels of polyUb-p53 may be regulated by autophagy. Thus, we examined whether p53 was taken up by autophagosomes. To test this hypothesis, we performed IF for both p53 and LC3 splice variant B (LC3B). In control cells, LC3B was mainly distributed in the perinuclear area in the cytoplasm but became aggregated after Cd exposure and completely colocalized with p53 (Fig. 5a). The potential interaction between p53 and LC3B was examined using IP analysis in cells treated with Cd for 6 and 12 h using an anti-p53 antibody followed by immunoblotting using an anti-LC3B antibody (Fig. 5b, c). These results revealed that p53 interacted with LC3B. Collectively, these results indicate that the stabilization and subcellular localization of p53 are regulated by autophagy in Cd-exposed MES13 cells.

### Cd resistance is associated with inhibition of polyUb-p53 and polyUb-p62

**Fig. 6:**
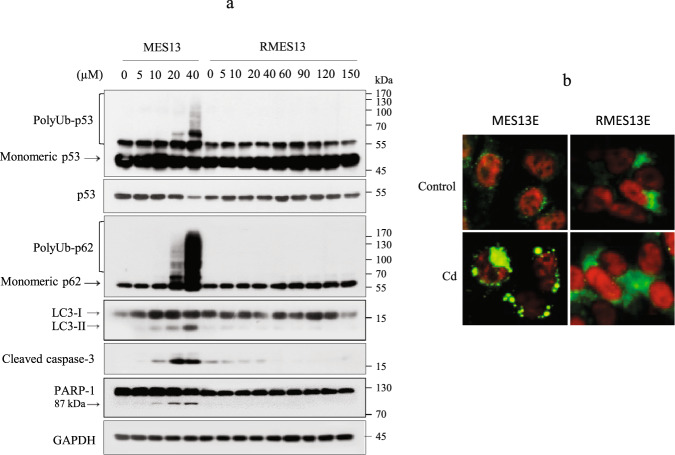
Quantification of polyUb-p53 and polyUb-p62 in Cd-resistant cells (RMES13E). **a** MES13E and RMES13E cells were exposed to increasing concentrations of Cd for 12 h. Lysates were subjected to immunoblotting for the indicated proteins. GAPDH was used as the loading control. *n* = 3. **b** RMES13E cells cultured on coverslips were treated with 100 µM Cd and fixed, and then IF was performed for both p53 (red) and p62 (green). The nuclei were counterstained with Hoechst 33342 (blue). Scale bar = 25 µm.

To investigate the role of polyUb-p53 and polyUb-p62 in kidney disease, MES13E cells were exposed to gradually increasing Cd concentrations to establish a Cd-resistant MES13E cell line (RMES13E) after multiple selections. These cells showed ~6-fold higher resistance to Cd than wild-type MES13E cells, which was analyzed using MTT analysis (Supplementary Fig. 6a). Cd-treated RMES13E cells exhibited complete inhibition of polyUb-p62 and polyUb-p53 with Cd exposure of up to 150 µM, while monomer-p62 and monomer-p53 levels were upregulated. Consistent with these results, autophagic flux and caspase-3-mediated PARP-1 cleavage were inhibited in Cd-treated RMES13E cells (Fig. 6a). In Cd-exposed RMES13E cells, the nuclear p53 signal was higher than those in control cells and wild-type cells. In addition, p62 showed a strong and diffuse staining pattern in the cytoplasm and was not aggregated in RMES13E cells exposed to 100 µM Cd (Fig. 6b).

Next, we investigated whether autophagy suppression in Cd-exposed RMES13E cells was due to inhibition of LC3B fluctuation and whether it was associated with the inhibition of polyUb-p53 and polyUb-p62. To this end, autophagy was blocked in RMES13E cells by using BaF1, and then cells were treated with two different concentrations of Cd: the IC_50_ concentration (23 µM) of wild-type cells and a high concentration (100 µM). BaF1 pretreatment of RMES13E cells exposed to 23 µM Cd slightly upregulated monomer-p62 and did not induce polyUb-p62, leading to the accumulation of LC3-II at levels similar to those after treatment with BaF1 alone. RMES13E cells treated with 100 µM Cd showed upregulation of monomer-p62 and monomer-p53 and slight upregulation of polyUb-p62 without induction of polyUb-p53. BaF1 pretreatment before addition of 100 µM Cd resulted in slight accumulation of polyUb-p62 and LC3-II to greater levels than those elicited by BaF1 alone. However, the level of LC3-II after BaF1 treatment in RMES13E cells was very low compared to that in wild-type cells. These results suggest that Cd-resistant cells have a reduced ability to induce polyUb-p62, polyUb-p53, and LC3-II expression in response to Cd (Supplementary Fig. 6b). Next, we examined the expression of p53 and p62 in renal cell carcinoma (RCC) cell lines, including ACHN and Caki-I. In ACHN cells, p53 expression was slightly decreased by more than 20 µM Cd, and polyubiquitination was not induced in response to Cd. ACHN cells expressed high levels of basal p62. The p62 level decreased with the Cd concentration, which led to LC3-II induction (Supplementary Fig. 7a), indicating that functional autophagy was induced in ACHN cells. Consistently, BaF1 treatment upregulated Cd-induced LC3-II, monomer-p62, and polyUb-p62 levels (Supplementary Fig. 7b). A low concentration of p53 was detected in the perinuclear area using IF, and p62 was exclusively localized in the cytoplasm with very strong and diffuse staining in control cells. The p62 signal was reduced, accumulated into a large aggregate, and partially colocalized with p53 in the cytoplasm after Cd exposure. Caki-I cells expressed high basal levels of p53 and p62, expressed small amounts of HMW-p62 upon exposure to Cd, and exhibited slightly induced autophagy in response to Cd treatment of up to 60 µM. In the control Caki-I cells, the staining patterns for p53 and p62 were similar to those in ACHN cells. p62 localization was punctate in the cells treated with Cd and partially colocalized with cytoplasmic p53. In both cell lines, p53 was not significantly punctate and was localized to the nucleus and cytoplasm after Cd exposure (Supplementary Fig. 7c). These results suggest that Cd resistance in normal kidney cells is closely related to the nuclear accumulation of monomer-p53 and the inhibition of polyUb-p53. Cancer cells repress polyUb-p53 in response to Cd, which may be due to Cd resistance.

### p53 expression in Cd-exposed mouse kidneys

**Fig. 7:**
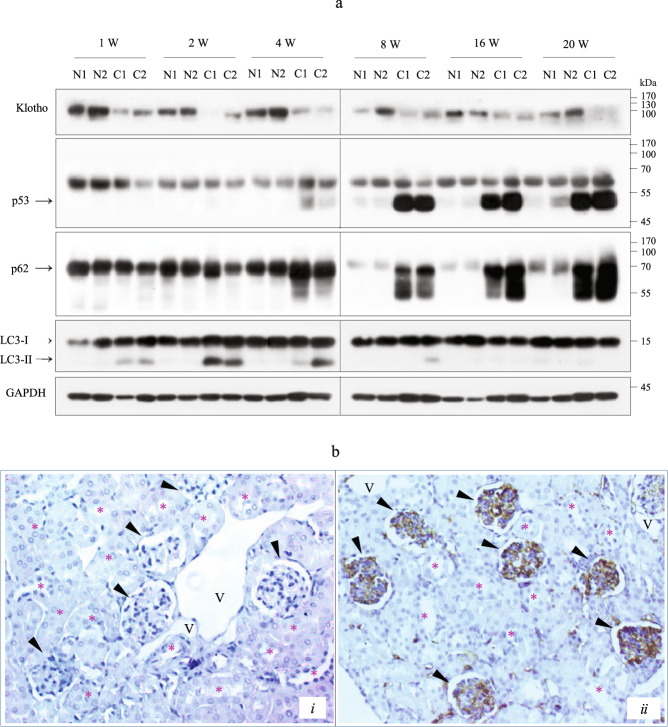
Expression of p53 and p62 in Cd-exposed mouse kidney tissues. **a** Mice were exposed to saline or Cd through i.p. injection. Kidney tissue lysates were analyzed for the indicated proteins by immunoblotting. GAPDH was used as the loading control. N, saline-injected normal control; C, Cd-injected mouse. **b** Immunohistochemical analysis of p53 in kidney tissues after 16 weeks of saline (i) and Cd (ii) injection in mice. Original magnification = ×200. Arrowheads = glomerulus, asterisks = tubules, V = vessels. Scale bar = 25 μm.

We examined whether polyUb-p53 and polyUb-p62 were associated with Cd-induced kidney damage in vivo. Mice were exposed to Cd via intraperitoneal (i.p.) injections for 20 weeks, as previously described^25^. Kidney tissues were collected at intervals for 20 weeks after Cd injection, and lysates were prepared for immunoblotting. The levels of klotho, a biomarker of chronic kidney disease, began to decline a week after Cd injection, but this molecule was continuously expressed in normal mice with only minor decreases, indicating that Cd caused kidney damage. During Cd injection, p53 levels began to increase at 4 weeks and continually increased throughout the 20 weeks. Similar to the p53 levels, the p62 levels began to increase at 4 weeks and then persistently increased up to 20 weeks. Within 4 weeks, polyUb-p53 and polyUb-p62 were detected at low levels in Cd-injected mouse kidney tissues (Supplementary Fig. 8). There was an inverse correlation between the increased monomer-p62 and LC3-II levels (Fig. 7a). To confirm p53 localization, IHC was performed using an anti-p53 antibody on kidney tissues from Cd- and saline-injected mice at 16 weeks. p53 expression was high in the glomeruli of Cd-injected mice but not in those of saline-injected controls (Fig. 7b-i, b-ii). There was no p53 signal in tubular epithelial cells or the negative control conditions, which consisted of incubation with the antibody dilution buffer instead of the anti-p53 antibody (data not shown). In addition, phospho-ERK, phospho-Akt, and Bcl-2 were significantly upregulated, and procaspase-3 cleavage was inhibited in kidney tissues from Cd-injected mice. Consistently, cleavage of PARP-1 was observed within 4 weeks. In addition, the endoplasmic reticulum stress-mediated protein CHOP was induced within 4 weeks in the control and Cd-injected kidney tissues and completely inhibited in the kidney tissue injected with Cd for 8 weeks or more (Supplementary Fig. 8). These results suggest that increased p53 expression in mesangial cells could be a marker for Cd resistance.

## Discussion

The present study showed that Cd exposure of MES13E cells expressing high basal levels of p53 in the nucleus induced polyubiquitination of p53, translocation and accumulation of p53 in the cytoplasm, and apoptosis. The expression pattern and posttranslational modification of p53, including monomer-p53 and polyUb-p53, were specific to Cd exposure in MES13E cells. The cytoplasmic accumulation of polyUb-p53 was regulated by polyUb-p62 and autophagy. Suppression of polyUb-p53, polyUb-p62, and autophagy was observed in Cd-resistant MES13E cells and in kidney tissues from mice that were continually injected with Cd. Therefore, polyUb-p53 may be an early biomarker of Cd-induced damage in mesangial cells, and a dysregulated accumulation of monomer-p53 may contribute to tumor development in mesangial cells.

Many studies have reported that p53, a critical tumor suppressor protein, plays an important role in Cd-induced cytotoxicity via apoptotic cell death^28^. p53 accumulation is caused by a reduced UBE2D level in the kidneys and proximal tubular cells^6,7^. In addition, in normal human prostate cells, wild-type p53 plays an important role in Cd-induced apoptosis. Thus, prostate cancer cells with insufficient p53 expression or those expressing mutant p53 are resistant to Cd-induced apoptosis^29^. In contrast, a protective role of p53 against Cd-induced cytotoxicity has been reported in A549, HEK293, and HCT116 cells^30^. In that study, Cd exposure downregulated p53 at the transcriptional level in wild-type HCT116 (p53^+/+^) cells. This response was less sensitive in p53-deficient (p53^−/−^) HCT116 cells, suggesting that p53 may be involved in cell survival. This discrepancy in the role of p53 may be due to the subcellular localization, stress conditions, and cell type.

Previous studies have demonstrated that the role of p53 in Cd-induced kidney damage is attributable to the levels of the p53 protein. However, the subcellular localization of p53 also plays a critical role in regulating apoptosis and autophagy. Nuclear p53 regulates many apoptosis-related proteins at the transcriptional level, including Bax, PUMA, MDM2, Fas/CD95, and Noxa. Cytoplasmic p53 induces apoptosis in a transcription-independent manner by either directly triggering mitochondrial outer membrane permeabilization or interactions with antiapoptotic proteins^31^. Nuclear p53 is associated with autophagy induction in a DNA damage-dependent manner, and cytoplasmic p53 is involved in autophagy inhibition^23^. However, the effect of the subcellular localization of p53 in Cd-induced kidney damage has not been studied. Cd cytotoxicity in the kidneys has been attributed to increased apoptotic and autophagic cell death^32–35^. Most studies on Cd toxicity have focused on tubular cells due to their reabsorption properties; in contrast, not much attention has been given to mesangial cells. Mesangial cells are important because they are in direct contact with capillary endothelial cells and can exchange substances with them. Furthermore, mesangial cells can effectively induce autophagy and apoptosis in response to Cd^26,33,34^. However, the involvement of p53 in both signaling pathways remains unclear.

Compared to steady-state conditions, under stress, the p53 protein is stabilized through various posttranslational modifications by several enzymes^3^. MES13E cells have high levels of p53Ac and p-p53, which can help maintain the high basal levels of p53. The levels of p53Ac and p-p53 decreased during Cd exposure, suggesting that inhibition of posttranscriptional modifications helped regulate p53 stability. However, this hypothesis was not supported in further experiments. p53 was detected in monomeric and polyUb-conjugated forms in Cd-exposed MES13E cells. Upon treatment with various stressors, including H_2_O_2_, which can induce autophagy, polyUb-p53, and polyUb-p62 were not induced in MES13E cells (Supplementary Fig. 9a, b), indicating that polyUb of p53 and p62 is specific to Cd stimulation. In Cd-exposed MES13E cells, monomer-p53 levels decreased during Cd exposure, but polyUb-p53 persistently accumulated in the cytoplasm, suggesting that polyUb-p53 accumulation in the cytoplasm helped to avoid degradation. The levels of the p53 protein may be regulated by ubiquitin E3 ligases, including MDM2, which promotes p53 degradation by nuclear and cytoplasmic proteasomes, since MDM2 itself is also a target of the proteasome^36^. However, MDM2 levels were not changed by Cd exposure or proteasome inhibition, indicating that Cd-induced changes in p53 levels were independent of MDM2. The selective clearance of proteins requires ubiquitination for targeting; the targeted proteins are then cleared by autophagy or the proteasome^37^. MG132, a proteasome blocker and an autophagy inducer, was used to investigate whether p53 protein levels in Cd-exposed MES13E cells were dependent on the proteasome or autophagy. Treatment with MG132 with and without Cd increased the levels of proteasome targets, including p21 and polyUb proteins^38^. Unlike MG132 alone, MG132 responded differently to Cd depending on the concentration used. Low concentrations of MG132 (≤0.5 μM) upregulated monomer-p53 and polyUb-p53 in response to Cd, and high concentrations (1–5 μM) reduced the levels of both forms. In addition, polyUb-p62 and polyUb-p53 were upregulated during the constitutive expression of LC3-II, but a decrease in the LC3-II level was positively correlated with decreases in polyUb-p62 and polyUb-p53 levels (Fig. 2e, Supplementary Fig. 4a). These results indicate that the accumulation of polyUb-p53 in Cd-exposed cells may be regulated by polyUb-p62 and autophagy. In contrast, proteasome blockade by MG132 in autophagy-impaired MES13E cells did not increase the levels of polyUb-p53, polyUb-p62, and polyUb proteins induced by Cd treatment compared to MG132 treatment in wild-type cells. These results show that autophagy may play a critical role in the regulation of polyUb-p53 in response to Cd and that crosstalk between the proteasome and autophagy does not exist in Cd-exposed cells. In fact, suppressing autophagy by knocking down *ATG5* not only reduced the increase in Cd-induced polyUb-p53 and polyUb protein expression (data not shown). Pharmacological inhibition of autophagy by BaF1 further increased the Cd-induced cytoplasmic accumulation of polyUb-p53 and subsequently increased Cd-induced apoptosis. BaF1 can inhibit the fusion between autophagosomes and lysosomes, inhibiting autophagic flux that results in autophagosome accumulation in the cytosol and, consequently, the accumulation of the polyUb-p62-cargo complex in the autophagosome. A reduced polyUb-p62 level resulted in p53 accumulation in the nucleus, indicating that p62 was critical for the cytoplasmic translocation of p53. It has been reported that p62 acetylation is required for the formation of the p62 body^39^. However, we did not find Cd-induced p62 acetylation by using deacetylase inhibitors, including trichostatin A and nicotinamide (data not shown). Therefore, our study indicated that the cytoplasmic accumulation of polyUb-p53 was regulated by the cooperative action of polyUb-p62 and autophagy.

This study shows that nuclear monomer-p53 protects cells against apoptosis, whereas cytoplasmic polyUb-p53 may help promote apoptosis. Resistance to Cd is caused by suppression of apoptosis, which may contribute to various diseases, including cancer^40^. We found that Cd resistance in MES13E cells was associated with polyUb-p53 inhibition and monomer-p53 accumulation. Similar results were obtained in kidney tissues from mice that were continually injected with Cd, with high levels of monomer-p53 localized in the glomeruli. Therefore, these results support our hypothesis for the role of p53 in wild-type MES13E cells. Furthermore, RMES13E cells had high levels of phospho-Akt (data not shown), and the kidney tissues from mice that were continually exposed to Cd had upregulated phospho-Akt, phospho-ERK, and antiapoptotic Bcl-2 levels and decreased cleavage of procaspase-3 and PARP-1 (Supplementary Fig. 8), which can contribute to tumor development^41–43^. These results suggest that inhibition of polyUb-p53 may be involved in protecting normal cells against Cd-induced apoptosis but may promote tumor formation under certain conditions. Indeed, ACHN and Caki-I cell lines had high IC_50_ values for Cd (~70 µM, data not shown) and did not express polyUb-p53 after Cd exposure. Functional autophagy was induced in ACHN cells at all Cd concentrations used, but polyUb-p53 was not induced. Although Caki-I cells exhibited formation of p62 puncta upon Cd exposure, there was no change in the p53 protein level or significant induction of autophagy. Therefore, the repression of polyUb-p53 in kidney cancer cells may be related to some degree to Cd resistance. We examined the effects of ≤60 µM Cd in both cell lines. The role of p53 in malignant cells requires further study.

It is unclear how nuclear p53 translocates to the cytoplasm in response to Cd exposure. We observed that Cd exposure led to increased expression of the autophagy adapter p62, which was detected in monomeric and polyUb-conjugated forms, similar to the pattern observed for p53 during Cd exposure (Supplementary Fig. 3b, c), suggesting a possible interaction between the two proteins. In this study, we observed polyUb-p53 interaction and colocalization with polyUb-p62 in the cytoplasm. However, it remains unclear whether p53 directly or indirectly binds to p62. p62 was localized in the perinuclear area in untreated control MES13E cells. After Cd exposure, polyUb-p62 was predominantly localized in the cytoplasm in puncta of various sizes. p62 is an adapter for ubiquitinated cargo that delivers the cargo to autophagosomes or proteasomes for degradation and is also degraded alongside the cargo via autophagy^44^. In response to Cd, p53 aggregates in the perinuclear area before migrating to the cytoplasm. This suggests that the nuclear export of p53 requires posttranscriptional modifications, such as polyubiquitination, facilitating the targeting of p53 by p62 in the nuclear membrane. This was shown by silencing of *p62*, which suppressed polyUb-p53 and restored monomer-p53 localization in the nucleus. In contrast, p53 knockdown repressed polyUb-p62 expression.

It is still unclear whether nuclear p53 interacts with p62. p62 has two nuclear localization signals (NLSs) and a nuclear export signal (NES) and is involved in protein quality control via nucleocytoplasmic trafficking^44^. In addition, p62 is a major component of nucleoporins, which consist of the nuclear pore complex (NPC), a tunnel that facilitates nucleocytoplasmic shuttling across the nuclear membrane^45^. Knockdown of the translocated promoter region (Tpr), a component of NPC, increases p53 nuclear accumulation, indicating that the cytoplasmic translocation of p53 is mediated by NPC^46^. In addition, the nuclear export of p53 can be regulated by the nuclear export receptor chromosomal region maintenance 1 (CRM1), which binds to the NES of cargo molecules and cargo receptor p62^47,48^. Kang et al.^18^ reported that p62 indirectly interacts with p53 via TGase 2 under starvation conditions and transfers p53 into autophagosomes for degradation, protecting the cells against apoptosis in renal cell carcinoma. Therefore, nuclear p53 may be transferred by CRM1 to the nuclear pore, where p53 may be targeted by polyUb-p62 and then delivered into autophagosomes. However, in the present study, we did not evaluate the interaction among CRM1, p53, and p62; this remains a topic for future studies.

In conclusion, our findings reveal that Cd exposure leads to polyubiquitination, cytoplasmic translocation, and transient accumulation of p53, which can promote cell death through apoptosis. This process may be regulated by polyUb-p62 and autophagy. Therefore, suppression of polyUb-p62 and autophagy promotes the nuclear accumulation of p53 in kidney mesangial cells. These results suggest that polyUb-p53 may be an early biomarker for Cd cytotoxicity in mesangial cells and that dysregulated accumulation of monomer-p53 may play a critical role in tumor development. The intracellular localization of p53 demonstrates its potential for use as a clinical indicator of Cd-induced cytotoxicity and cancer development.

## Supplementary information

Supplementary figure

## References

[1] Agency for Toxic Substances and Disease Registry Toxicological Profile for Cadmium. *Agency for Toxic Substances and Disease Registry*. Atlanta, Georgia, 1–397 (1999).

[2] Järup L, Akesson A (2009). Current status of cadmium as an environmental health problem. Toxicol. Appl. Pharmacol..

[3] Vousden KH, Prives C (2009). Blinded by the light: the growing complexity of p53. Cell.

[4] Appella E, Anderson CW (2000). Signaling to p53: breaking the posttranslational modification code. Pathol. Biol..

[5] Hock AK, Vousden KH (2014). The role of ubiquitin modification in the regulation of p53. Biochim. Biophys. Acta.

[6] Lee, J. Y. et al. Accumulation of p53 via down-regulation of UBE2D family genes is a critical pathway for cadmium-induced renal toxicity. *Sci. Rep*. 10.1038/srep21968 (2016). 10.1038/srep21968 PMC4766413 26912277

[7] Tokumoto M (2016). Cadmium toxicity is caused by accumulation of p53 through the down-regulation of Ube2d family genes in vitro and in vivo. J. Toxicol. Sci..

[8] Fujiki, K., Inamura, H. & Matsuoka, M. Detrimental effects of Notch1 signaling activated by cadmium in renal proximal tubular epithelial cells. *Cell Death Dis*. 10.1038/cddis.2014.339 (2014). 10.1038/cddis.2014.339 PMC4454314 25118938

[9] Xie J, Shaikh ZA (2006). Cadmium induces cell cycle arrest in rat kidney epithelial cells in G2/M phase. Toxicology.

[10] Mihara M (2003). p53 has a direct apoptogenic role at the mitochondria. Mol. Cell.

[11] Mizushima N, Komatsu M (2011). Autophagy: renovation of cells and tissues. Cell.

[12] Fujishiro H, Liu Y, Ahmadi B, Templeton DM (2018). Protective effect of cadmium-induced autophagy in rat renal mesangial cells. Arch. Toxicol..

[13] Liu, G. et al. Beclin-1-mediated autophagy protects against cadmium-activated apoptosis via the Fas/FasL pathway in primary rat proximal tubular cell culture. *Sci. Rep*. 10.1038/s41598-017-00997-w (2017). 10.1038/s41598-017-00997-w PMC5430518 28428545

[14] Luo T (2018). ERK1/2 MAPK promotes autophagy to suppress ER stress-mediated apoptosis induced by cadmium in rat proximal tubular cells. Toxicol. Vitr..

[15] Wang SH, Shih YL, Kuo TC, Ko WC, Shih CM (2009). Cadmium toxicity toward autophagy through ROS-activated GSK-3beta in mesangial cells. Toxicol. Sci..

[16] Yang LY, Wu KH, Chiu WT, Wang SH, Shih CM (2009). The cadmium-induced death of mesangial cells results in nephrotoxicity. Autophagy.

[17] Guo JY, Xia B, White E (2013). Autophagy-mediated tumor promotion. Cell.

[18] Kang, J. H. et al. Renal cell carcinoma escapes death by p53 depletion through transglutaminase 2-chaperoned autophagy. *Cell Death Dis*. 10.1038/cddis.2016.14 (2016). 10.1038/cddis.2016.14 PMC4823929 27031960

[19] White E (2015). The role for autophagy in cancer. J. Clin. Invest..

[20] Budanov AV, Karin M (2008). p53 target genes sestrin1 and sestrin2 connect genotoxic stress and mTOR signaling. Cell.

[21] Crighton D (2006). DRAM, a p53-induced modulator of autophagy, is critical for apoptosis. Cell.

[22] Green DR, Kroemer G (2009). Cytoplasmic functions of the tumour suppressor p53. Nature.

[23] Tasdemir E (2008). A dual role of p53 in the control of autophagy. Autophagy.

[24] Kriz W, Elger M, Lemley K, Sakai T (1990). Structure of the glomerular mesangium: a biomechanical interpretation. Kidney Int. Suppl..

[25] Kim HR (2015). Transcriptional regulation, stabilization, and subcellular redistribution of multidrug resistance-associated protein 1 (MRP1) by glycogen synthase kinase 3αβ: novel insights on modes of cadmium-induced cell death stimulated by MRP1. Arch. Toxicol..

[26] So KY, Park BH, Oh SH (2021). Cytoplasmic sirtuin 6 translocation mediated by p62 polyubiquitination plays a critical role in cadmium-induced kidney toxicity. Cell Biol. Toxicol..

[27] Kubbutat MH, Jones SN, Vousden KH (1997). Regulation of p53 stability by Mdm2. Nature.

[28] Fujiwara Y, Lee JY, Tokumoto M, Satoh M (2012). Cadmium renal toxicity via apoptotic pathways. Biol. Pharm. Bull..

[29] Aimola, P. et al. Cadmium induces p53-dependent apoptosis in human prostate epithelial cells. *PLoS ONE*10.1371/journal.pone.0033647 (2012). 10.1371/journal.pone.0033647 PMC3308998 22448262

[30] Ravindran G, Chakrabarty D, Sarkar A (2016). Cell specific stress responses of cadmium-induced cytotoxicity. Anim. Cells Syst..

[31] Pu T, Zhang XP, Liu F, Wang W (2010). Coordination of the nuclear and cytoplasmic activities of p53 in response to DNA damage. Biophys. J..

[32] Liu Y, Templeton DM (2007). Cadmium activates CaMK-II and initiates CaMK-II-dependent apoptosis in mesangial cells. FEBS Lett..

[33] Wang SH, Shih YL, Ko WC, Wei YH, Shih CM (2008). Cadmium-induced autophagy and apoptosis are mediated by a calcium signaling pathway. Cell Mol. Life Sci..

[34] Wang SH, Shih YL, Kuo TC, Ko WC, Shih CM (2009). Cadmium toxicity toward autophagy through ROS-activated GSK-3beta in mesangial cells. Toxicol. Sci..

[35] Liu F (2017). Cadmium disrupts autophagic flux by inhibiting cytosolic Ca^2+^-dependent autophagosome-lysosome fusion in primary rat proximal tubular cells. Toxicology.

[36] Eischen CM, Lozano G (2014). The Mdm network and its regulation of p53 activities: a rheostat of cancer risk. Hum. Mutat..

[37] Korolchuk VI, Menzies FM, Rubinsztein DC (2010). Mechanisms of cross-talk between the ubiquitin-proteasome and autophagy-lysosome systems. FEBS Lett..

[38] Blagosklonny MV, Wu GS, Omura S, el-Deiry WS (1996). Proteasome-dependent regulation of p21WAF1/CIP1 expression. Biochem. Biophys. Res. Commun..

[39] You Z (2019). Requirement for p62 acetylation in the aggregation of ubiquitylated proteins under nutrient stress. Nat. Commun..

[40] Hartwig A (2010). Mechanisms in cadmium-induced carcinogenicity: recent insights. Biometals.

[41] Kirkin V, Joos S, Zörnig M (2004). The role of Bcl-2 family members in tumorigenesis. Biochim. Biophys. Acta.

[42] Testa JR, Bellacosa A (2001). AKT plays a central role in tumorigenesis. Proc. Natl Acad. Sci. USA.

[43] Guo YJ (2020). ERK/MAPK signalling pathway and tumorigenesis (Review). Exp. Ther. Med..

[44] Liu, W. J. et al. p62 links the autophagy pathway and the ubiqutin-proteasome system upon ubiquitinated protein degradation. *Cell Mol. Biol. Lett*. 10.1186/s11658-016-0031-z (2016). 10.1186/s11658-016-0031-z PMC5415757 28536631

[45] Knockenhauer KE, Schwartz TU (2016). The nuclear pore complex as a flexible and dynamic gate. Cell.

[46] Funasaka, T., Tsuka, E. & Wong, R. W. Regulation of autophagy by nucleoporin Tpr. *Sci. Rep*. 10.1038/srep00878 (2012). 10.1038/srep00878 PMC3501823 23170199

[47] Kanai M (2007). Inhibition of Crm1-p53 interaction and nuclear export of p53 by poly(ADP-ribosyl)ation. Nat. Cell Biol..

[48] Thakar K, Karaca S, Port SA, Urlaub H, Kehlenbach RH (2013). Identification of CRM1-dependent nuclear export cargos using quantitative mass spectrometry. Mol. Cell. Proteom..

