# Supplementary material for: The role of autophagy in cadmium-induced acute toxicity in glomerular mesangial cells and tracking polyubiquitination of cytoplasmic p53 as a biomarker
Source: Exp Mol Med. 2022 May 27;54(5):685–96. doi: 10.1038/s12276-022-00782-4 (PMC9166781; doi:10.1038/s12276-022-00782-4)
Supplement: Supplementary file 1 — Supplementary figure [file 12276_2022_782_MOESM1_ESM.pdf]

Supplementary Fig. 1

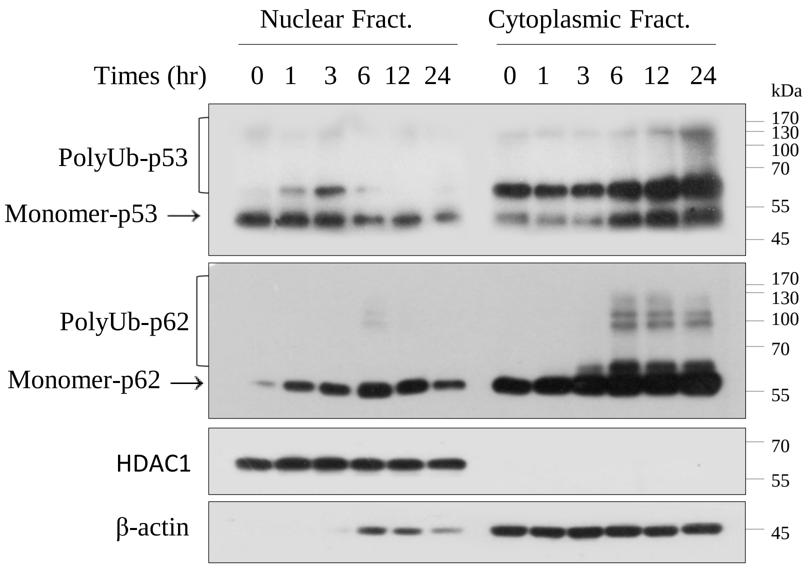

MES13E cells were washed with PBS, resuspended in hypotonic buffer [20 mM HEPES-KOH, pH 7.0, 10 mM KCl, 1.5 mM $MgCl_2$, 1 mM sodium EDTA, 1 mM EGTA, 250 mM sucrose (Sigma-Aldrich, S0389)] supplemented with cOmplete Mini EDTA-free protease inhibitor mixture (Roche Applied Science). The cells were homogenized by passing through a needle, followed by centrifugation at 800 g for 4 minutes. The supernatant and nuclear-enriched insoluble pellet were used as cytoplasmic and nuclear fractions, respectively. The purities of the isolated fractions were validated by immunoblotting for specific protein markers, such as histone deacetylase 1 (HDAC1) and β-actin for nuclear and cytoplasmic compartments, respectively.

Supplementary Fig. 2

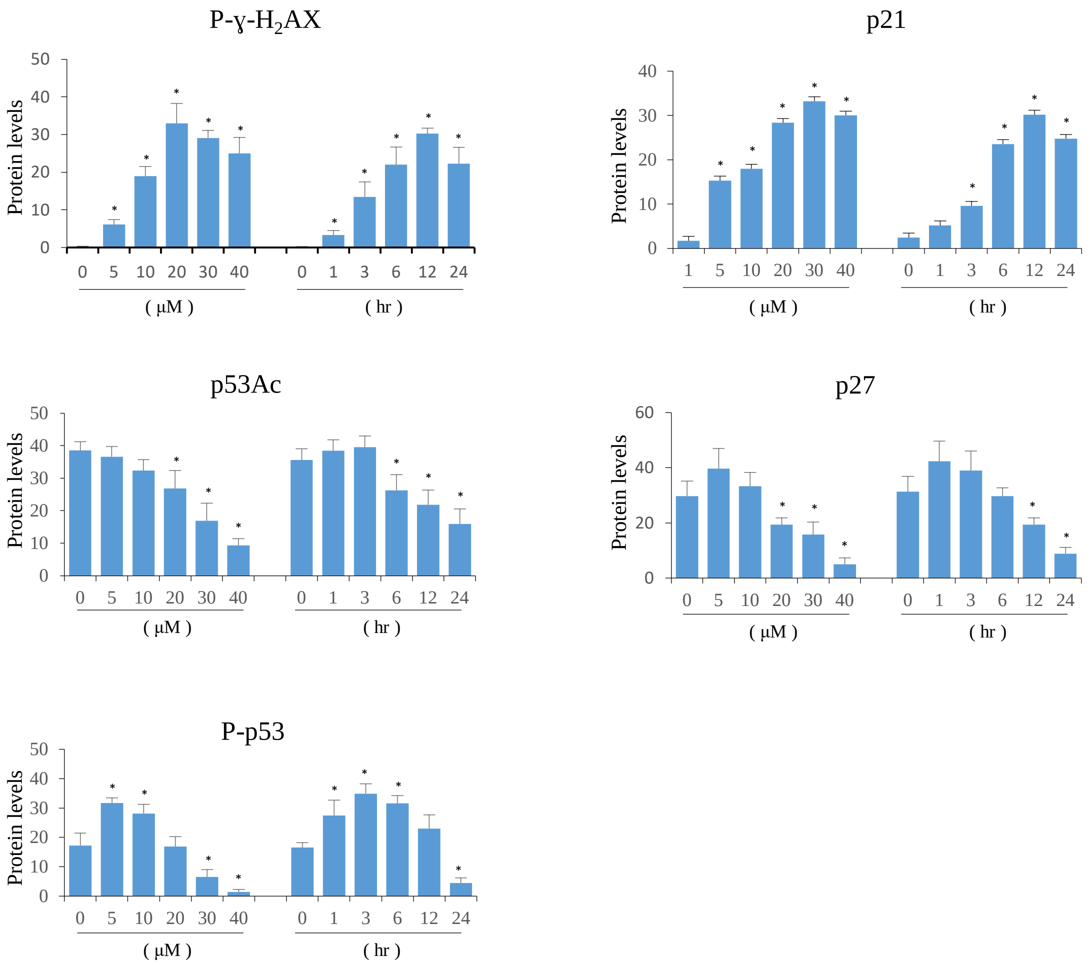

Protein quantification from Fig.1d was measured in optical density units using Image J program (National Institutes of Health, Bethesda, USA) and normalised to the corresponding sample expression of β-actin. n=3, Data were analyzed by *t*-student*t*-test. A value of $^{*}p< 0.05$ versus control was considered statistically significant.

Supplementary Fig. 3a-c

3a

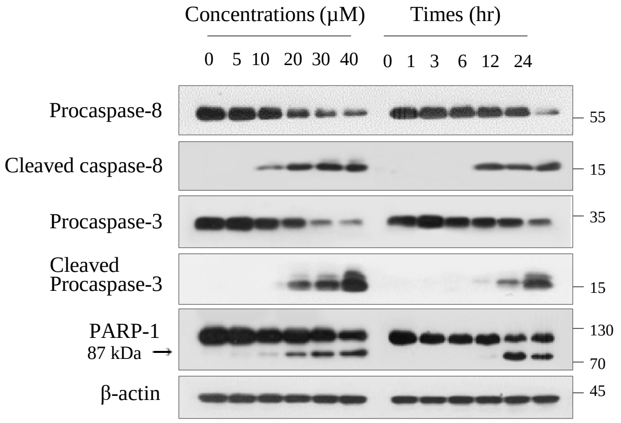

3b

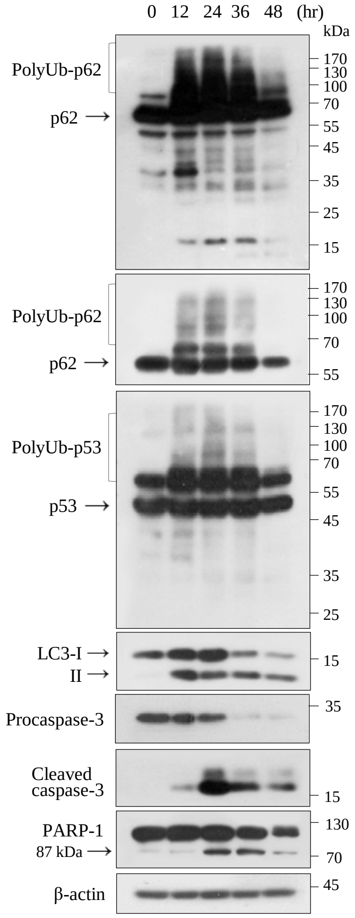

3c

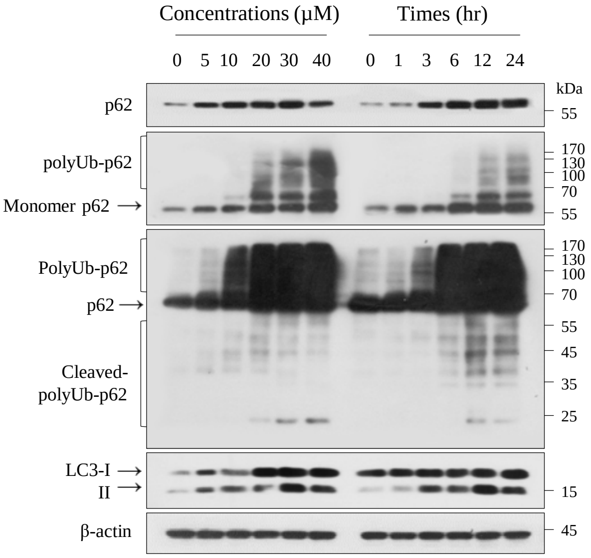

(**3a, c**) MES13E cells were treated with increasing Cd concentrations for 18 h or with 23 μM Cd up to 24 h , harvested, lyzed, and immunoblotted for the autophagy- and apoptosis-related proteins. (**3b**) MES13E cells were exposed to Cd (20 μM) up to 48 h. The expression pattern polyUb-p62 and monomer-p62, polyUb-p53 and apoptosis-related proteins were analyzed by immunoblotting. β-actin was used as the loading control.

Supplementary Fig. 4a-c

4a

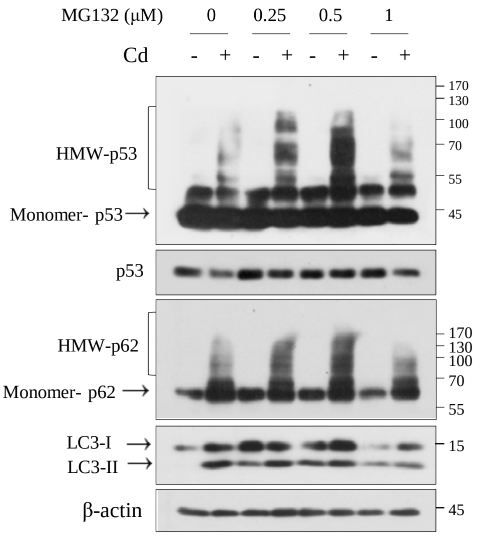

4b

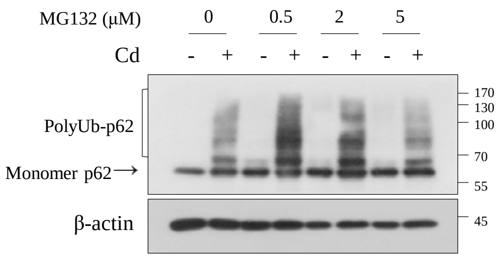

4c

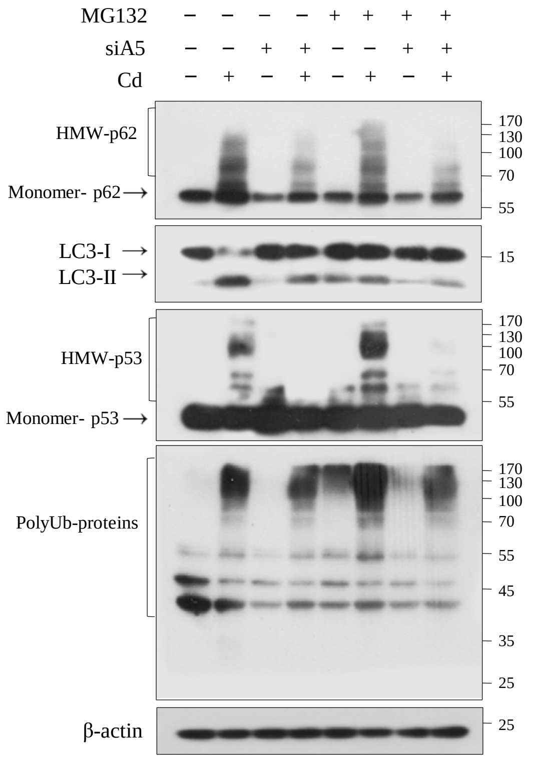

(**4a**) MES13 cells were pretreated with increasing concentrations of MG132 (0.25~1 μM) for 2 h and followed by Cd (23 μM) treatment for 12 h, harvested, lysed, and immunoblotted for p62. (**4b**) MES13 cells were pretreated with increasing concentrations of MG132 (0.5~5 μM) for 2 h and followed by Cd (23 μM) treatment for 12 h. β-actin was used as the loading control.

(**4c**) Cells transfected with negative control and *p62* siRNA were pretreated with MG132 (0.5 μM) or DMSO for 2 h and followed by Cd (23 μM) treatment for 12 h, harvested, lysed, and immunoblotted for indicated proteins. β-actin was used as the loading control.

Supplementary Fig. 5

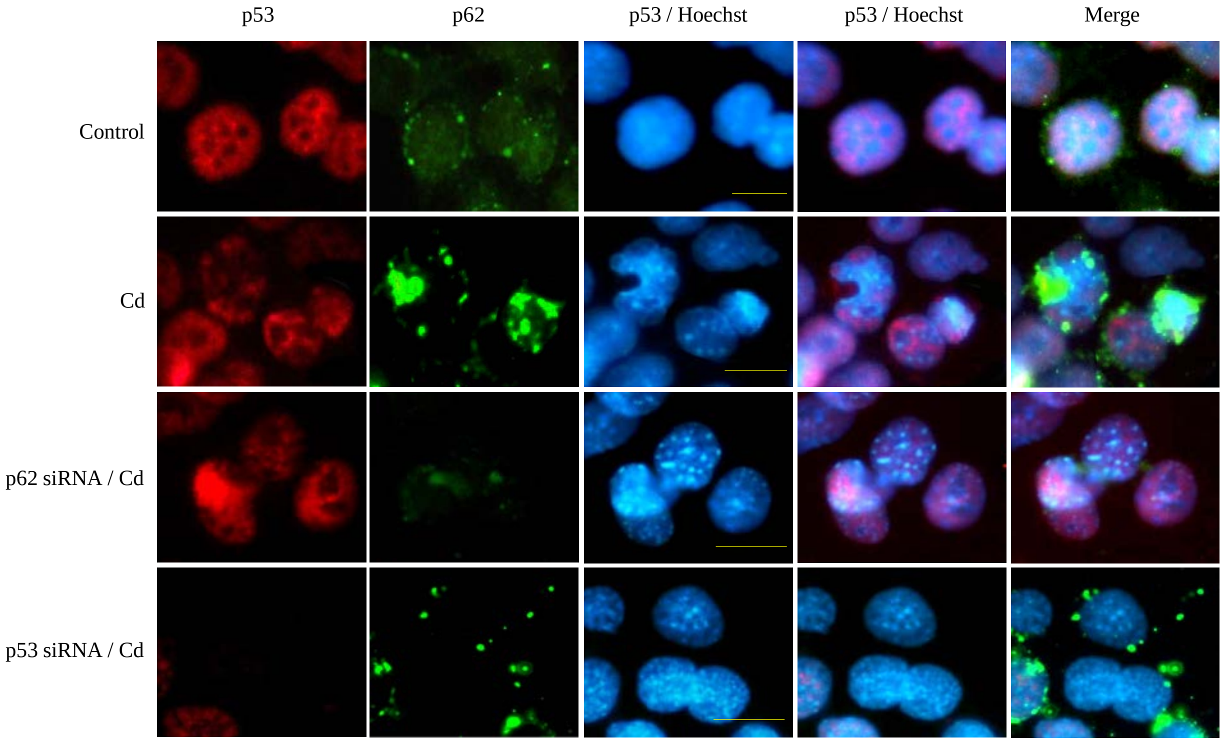

MES13E cells cultured on coverslips were treated as described in A and B, fixed, and then performed dual immunofluorescence labeling for both p53 (red) and p62 (green). Nuclei were counterstained with Hoechst 33342 (blue) and images were acquired with a fluorescence microscope. Arrows indicate aggregated p53 in the cytoplasm. Scale bar= 25 µm.

Supplementary Fig. 6a-b

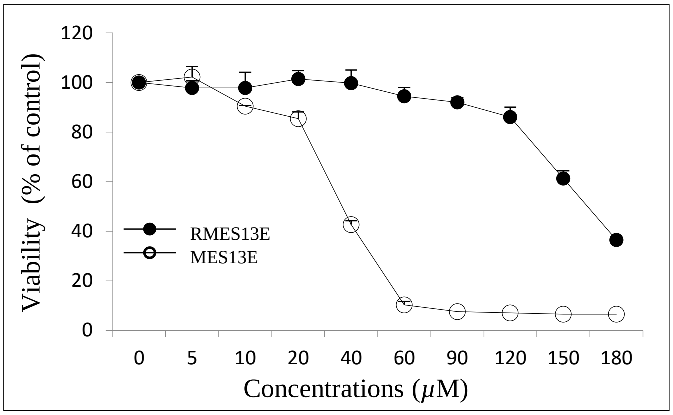

(**6a**) Cd-resistant MES13 cell line (RMES13) was established after several rounds of selection by exposing MES13 cells to gradually increasing Cd concentrations. To determine sensitivity to Cd, MES13E and RMES13E cells were exposed to increasing concentrations of Cd for 24 h and then subjected to an MTT assay. The IC_{50} values of Cd were about 23 μM and 160 μM, respectively. Data were expressed as the mean ± SD of fold-increase compared to the untreated control from three independent experiments performed in triplicate.

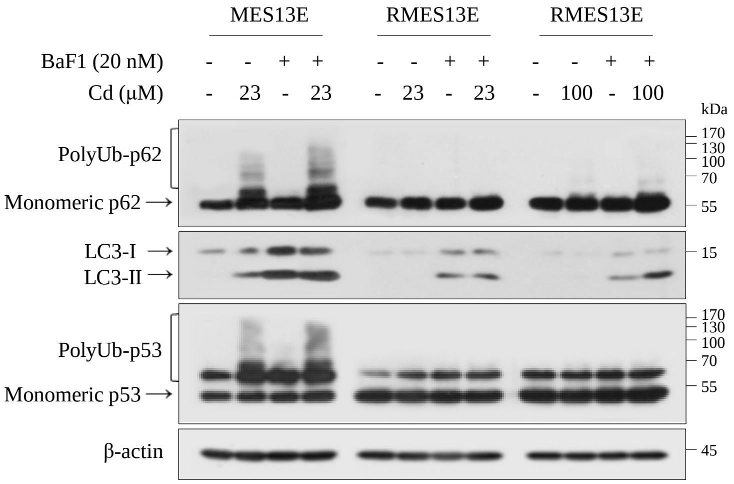

(**6b**) MES13E and RMES13E cells were exposed to 23 μM and 100 μM Cd for 12 h with or without pretreatment with BaF1 (20 nM) for 2 h. The lysates were subjected to immunoblotting for indicated proteins. β-actin was used as the loading control.

Supplementary Fig. 7a-c

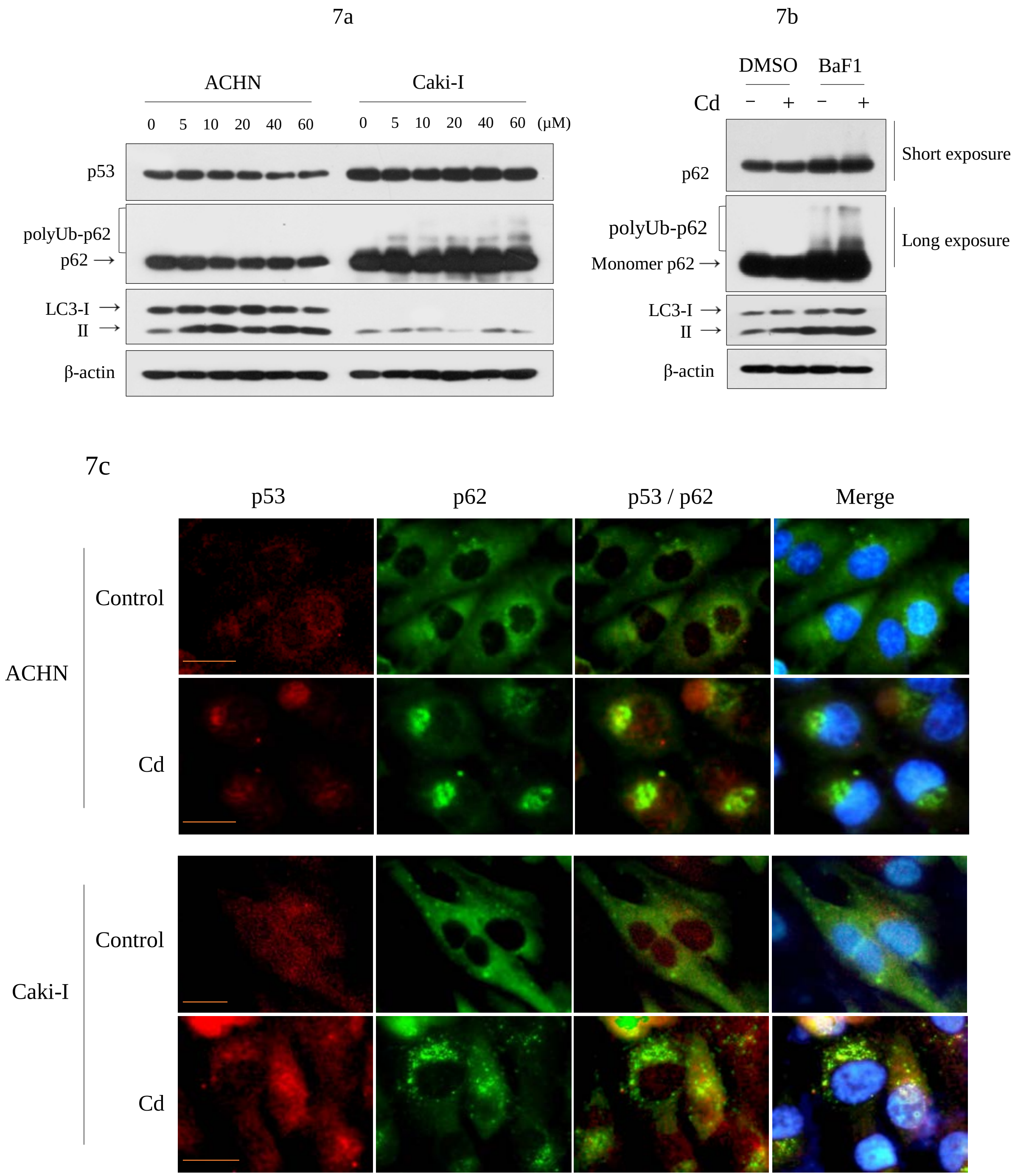

Responses of p53 and p62 proteins to Cd exposure in renal cancer cell lines. (**7a**) Cells were exposed to increasing concentrations of Cd, and analyzed for indicated proteins by immunoblotting. (**7b**) Cells were pretreated with BaF1 (20 nM) for 2 hr and continuously exposed with 40 μM Cd for 12 hr. (**7c**) Cells were cultured on a cover slip and exposed to 40 μM Cd, and performed immunocytochemistry for p53 (red) and p62 (green). Nuclei were stained with Hoechst 33342. Scale bar = 25 μm.

Supplementary Fig. 8

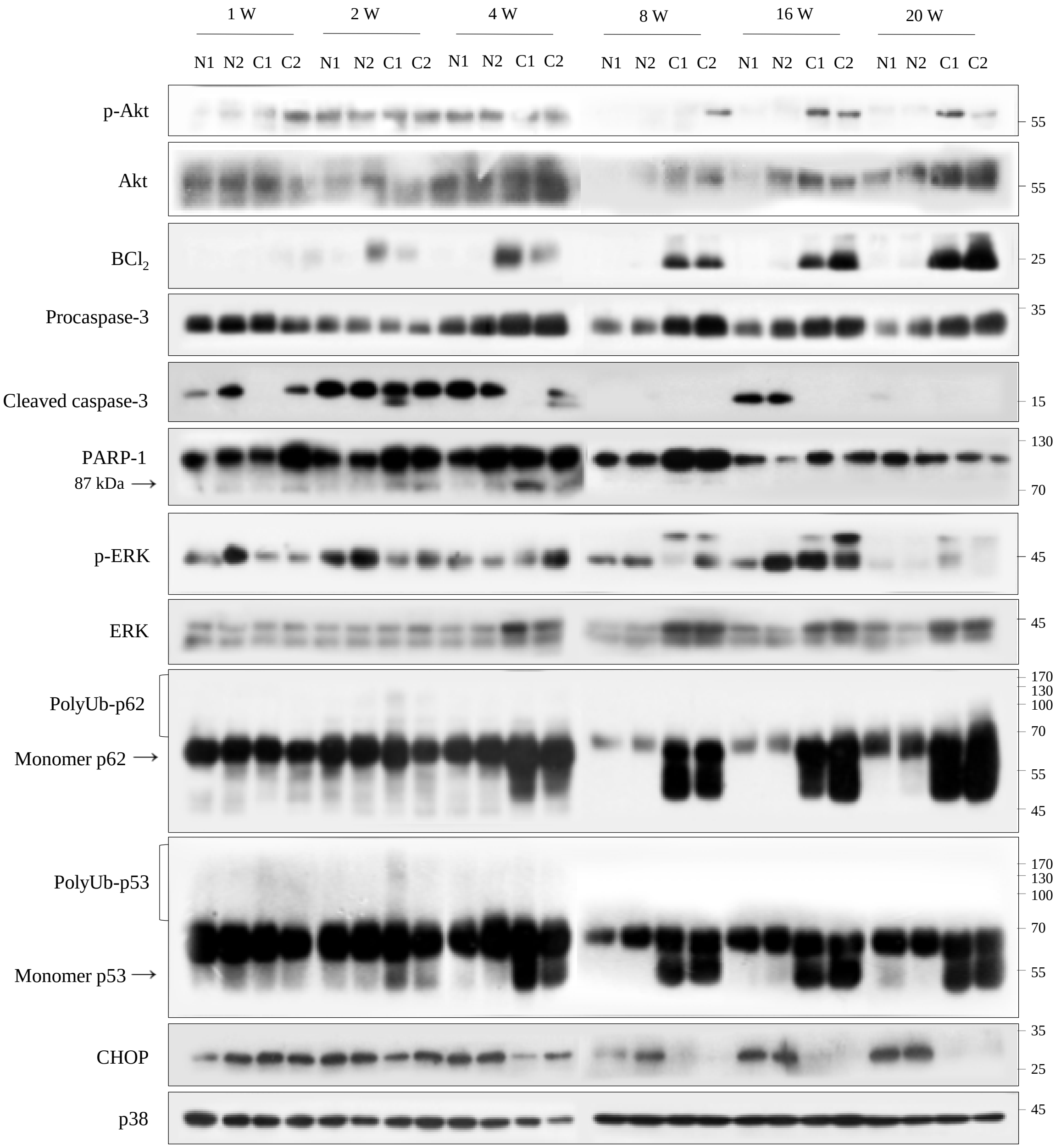

Mice were exposed to Cd by intraperitoneal (i.p.) injection for 20 weeks. Kidney tissue was collected at intervals for 20 weeks after Cd injection, and lysates were prepared for immunoblotting. Within 4 weeks, polyUb-p53 and polyUb-p62 were detected at low levels in Cd-injected mouse kidney tissues. Total p38 was used as the loading control. W, week; N, saline-injected; C, Cd-injected.

Supplementary Fig. 9a-b

9a

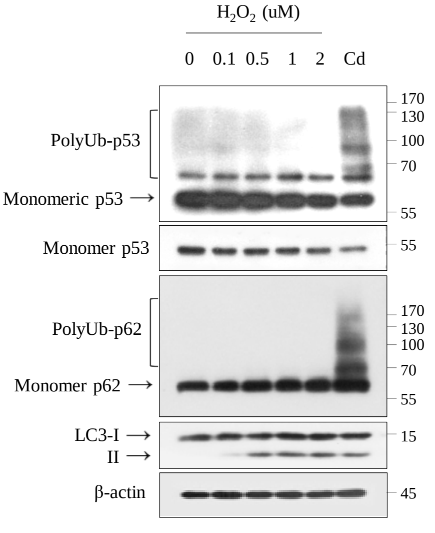

9b

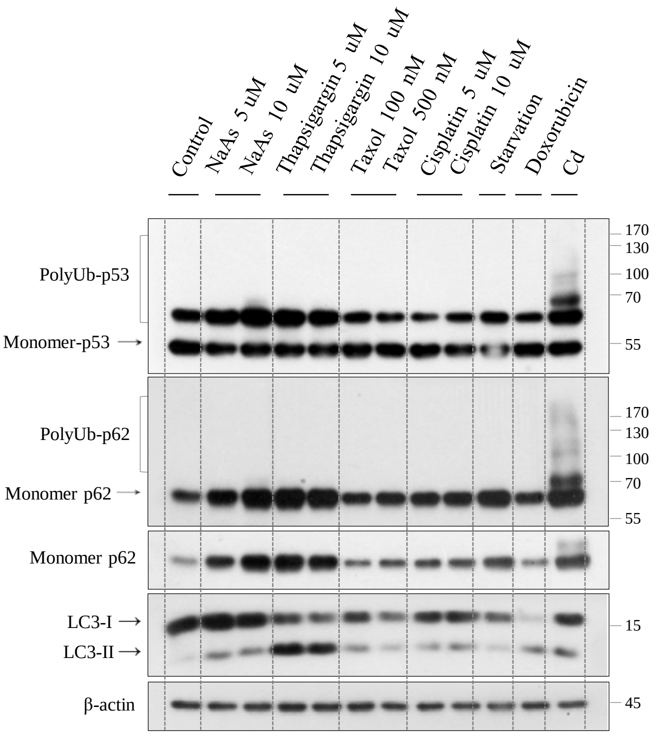

(**9a-b**) Expression pattern of p53 and p62 in response to different stressors. In order to further understand the expression pattern of p53 and p62, MES13E cells were exposed to various stimuli, conditions known to induce autophagy in renal cells. The cells were exposed to sodium arsenite, thapsigargin, taxol, cisplatin, starvation, and doxorubicin, and different concentrations of H_2O_2 for 12 h. Monomer-p53 increased by NaAs, thapsigargin, and starvation. Although p53 was decreased by taxol, cisplatin, and doxorubicin compared to control cells, it did not induce poly-ubiquitination of p53. Additionally, H_2O_2, a major cellular ROS source, caused decreasing p53, but polyUb-p53 and polyUb-p62 were not induced in MES13E cells. All stimulation excepting Cd expressed monomer –p53 and monomer-p62 only, indicating that the poly ubiquitination of p53 and p62 is specific for Cd in MES13E cells.
